# Polytyramine Film-Coated Single-Walled Carbon Nanotube Electrochemical Chemosensor with Molecularly Imprinted Polymer Nanoparticles for Duloxetine-Selective Determination in Human Plasma

**DOI:** 10.1021/acssensors.2c00124

**Published:** 2022-05-12

**Authors:** Jyoti, Teresa Żołek, Dorota Maciejewska, Edyta Gilant, Elzbieta Gniazdowska, Andrzej Kutner, Krzysztof R. Noworyta, Wlodzimierz Kutner

**Affiliations:** † Institute of Physical Chemistry, Polish Academy of Sciences, Kasprzaka 44/52, 01-224 Warsaw, Poland; ‡ Department of Organic Chemistry, Faculty of Pharmacy, Medical University of Warsaw, Banacha 1, 02-097 Warsaw, Poland; § Łukasiewicz Research Network−Industrial Chemistry Institute, Rydygiera 8, 01-793 Warsaw, Poland; ∥ Department of Bioanalysis and Drug Analysis, Faculty of Pharmacy, Medical University of Warsaw, Banacha 1, 02-097 Warsaw, Poland; ⊥ Faculty of Mathematics and Natural Sciences. School of Sciences, Cardinal Stefan Wyszynski University in Warsaw, Wóycickiego 1/3, 01-815 Warsaw, Poland

**Keywords:** molecularly imprinted polymer nanoparticle, nanoMIP, duloxetine, electrochemical chemosensor for duloxetine, single-walled carbon nanotube, SWCNT, polytyramine, molecular dynamics MIP modeling

## Abstract

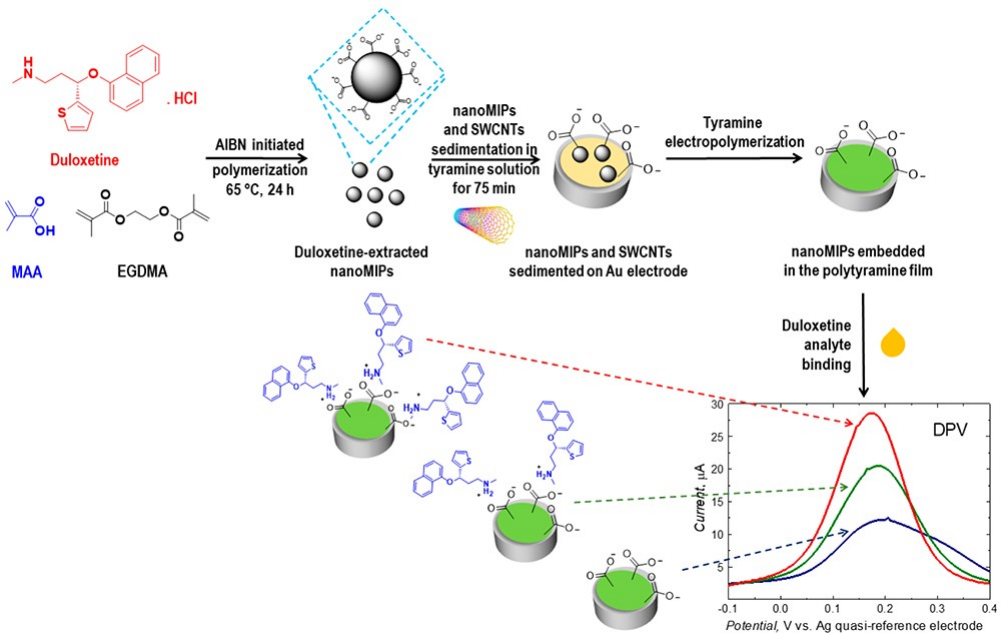

We devised, fabricated, and tested differential pulse voltammetry (DPV) and impedance spectroscopy (EIS) chemosensors for duloxetine (DUL) antidepressant determination in human plasma. Polyacrylic nanoparticles were synthesized by precipitation polymerization and were molecularly imprinted with DUL (DUL-nanoMIPs). Then, together with the single-walled carbon nanotube (SWCNT) scaffolds, they were uniformly embedded in polytyramine films, i.e., nanoMIPs-SWCNT@(polytyramine film) surface constructs, deposited on gold electrodes by potentiodynamic electropolymerization. These constructs constituted recognition units of the chemosensors. The molecular dynamics (MD) designing of DUL-nanoMIPs helped select the most appropriate functional and cross-linking monomers and determine the selectivity of the chemosensor. Three different DUL-nanoMIPs and non-imprinted polymer (nanoNIPs) were prepared with these monomers. DUL-nanoMIPs, synthesized from respective methacrylic acid and ethylene glycol dimethyl acrylate as the functional and cross-linking monomers, revealed the highest affinity to the DUL analyte. The linear dynamic concentration range, extending from 10 pM to 676 nM DUL, and the limit of detection (LOD), equaling 1.6 pM, in the plasma were determined by the DPV chemosensor, outperforming the EIS chemosensor. HPLC-UV measurements confirmed the results of DUL electrochemical chemosensing.

Major depressive disorder (MDD) has always been a long-standing global problem.^1,2^ It increases the risk of suicidal ideation and attempted suicide.^3^ Decreased concentrations in the central nervous system of neurotransmitters, such as serotonin (5-HT) and norepinephrine (NE), cause MDD. Selective serotonin reuptake inhibitors (SSRIs) have been well exploited, but because of their low selectivity and considerable side effects, new-generation antidepressants have been developed.^4^

Duloxetine (DUL) (Figure 1) is a selective serotonin–norepinephrine reuptake inhibitor (SNRI), effective in major depressive disorder,^1,3,5^ anxiety disorder,^6^ and fibromyalgia.^7^ DUL absorption begins 2 h after oral administration, and it reaches the maximum plasma concentration within ∼6 h.^6^ Several combinations of antidepressants have been tried to treat depressive illness, such as DUL-mirtazapine,^8^ DUL-amitriptyline,^9^ and antidepressants alone, such as venlafaxine.^10^ Patient-level post-hoc studies revealed no differences between DUL and other SSRIs in the sum score of the Hamilton Depression Rating Scale (HDRS-17-sum) in clinical trials.^11^

**Figure 1:**
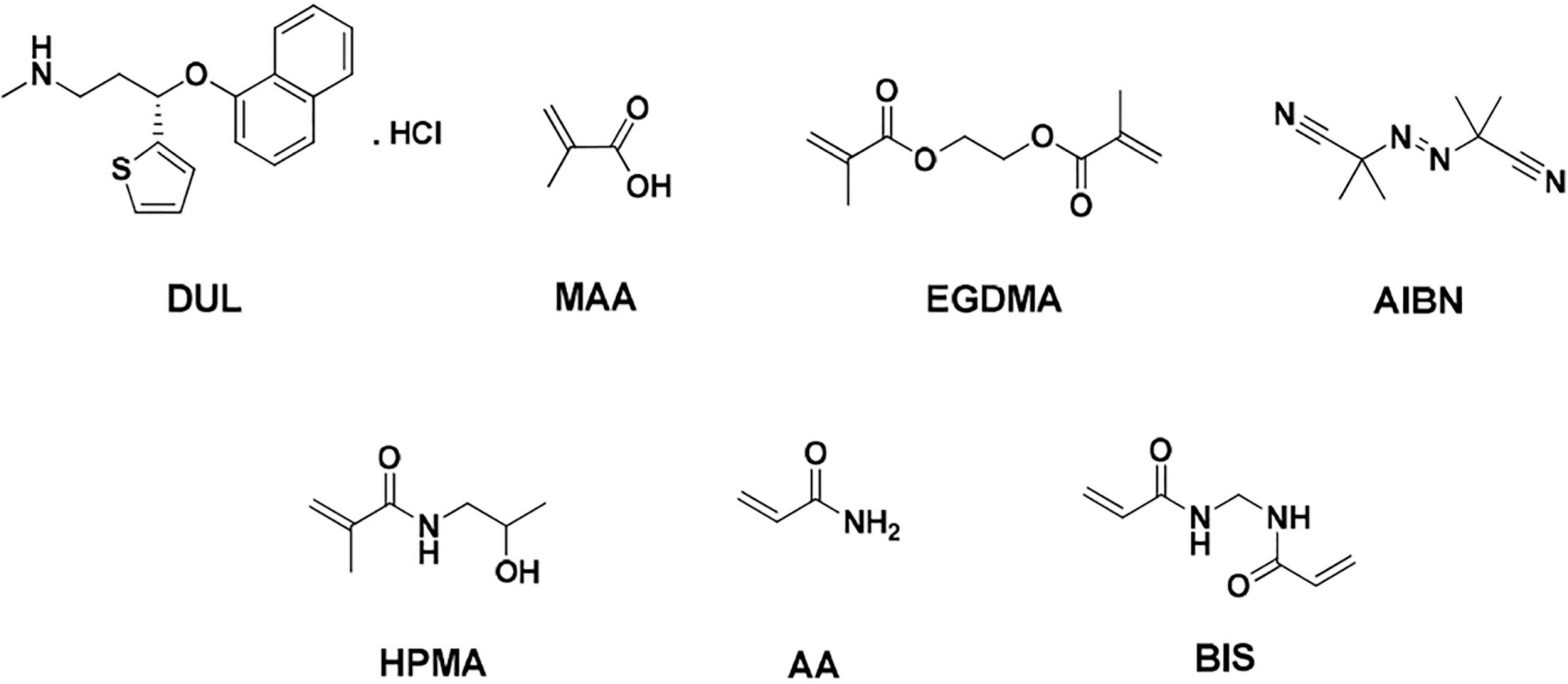
Structural formulas of duloxetine (DUL) analyte, methacrylic acid (MAA), acrylamide (AA), and *N*-2-hydroxypropyl methacrylamide (HPMA) functional monomers as well as ethylene glycol dimethyl acrylate (EGDMA), *N,N’*-methylenebis(acrylamide) (BIS) cross-linking monomers, and 2,2′-azobis(2-methylpropionitrile) (AIBN) initiator.

Analytical methods using HPLC^12−16^ and spectrofluorimetry^17,18^ are sensitive in determining DUL. However, the major drawbacks of typical analytical methods are their lengthy analysis time, low user friendliness, and expensive instrumentation needed. As an alternative, electroanalytical techniques are commonly employed to quantify drugs, environmental monitoring of industrial products, and medicinal chemicals, to name a few. The electrochemical characteristics of the target analyte often guide the measurement techniques selection. According to the analytical signal output nature, the measurement is the potential (*V*) in potentiometry, the current (*A*) in voltammetry and amperometry, the resistance (Ω) in impedimetry, the capacity (*F*) in capacitive impedimetry, and the conductivity (*S*) in conductometry. The current generated in amperometry or voltammetry can be used to quantify electroactive substances; however, nonelectroactive targets may alter the overall results. This alteration could indirectly be regulated and monitored using cyclic voltammetry (CV), differential pulse voltammetry (DPV), or electrochemical impedance spectroscopy (EIS) with an external redox probe.

Voltammetry is the most used electroanalytical technique due to its high sensitivity, low detection limit, easy operation, and simple instrumentation. Table S1 in the Supporting Information compares the analytical techniques and their parameters for DUL determination.

Molecularly imprinted polymer (MIP) operation is based on the “lock-and-key” principle of analyte recognition exploiting cavities left behind in the templated MIP upon template removal.^19,20^ Molecular imprinting is beneficial because it is an easy to develop, simple, highly selective, sensitive, and reconstructable procedure in which the cavities feature unique shapes, sizes, and recognizing functionalities of predefined orientations. Hence, they selectively recognize the analyte molecules via interactions with the analytes' binding sites.^20,21^ MIPs have widely been applied for separation^22^ and catalysis,^23^ as chemosensors’ recognizing units,^21^ etc.

Traditionally, imprinted polymers are synthesized by bulk polymerization requiring a template, different monomers, and a suitable initiator. Minimal or no porogen solvent is used to form a highly cross-linked monolith rigid polymer. This polymerization is followed by polymer block grinding and sieving, which is time consuming and causes the loss of a substantial amount of the MIP material. This rigid and condensed monolith structure hinders complete removal of the template, and some trapped “dead” sites in the imprinted polymer are left. Moreover, the bulk MIP grinding yields nonuniform particles where the recognizing sites are heterogeneously distributed.

Ye et al.^24^ first applied precipitation polymerization to MIPs’ preparation by synthesizing MIP beads in a submicrometer size range to overcome the above limitations. This polymerization is a heterogeneous polymerization that commences as a homogeneous reaction in a continuous phase, where the monomer and initiator are soluble in the solvent used. However, after initiation, the produced polymer progressively becomes insoluble and, hence, precipitates. This polymerization is relatively facile, resulting in evenly dispersed polymer micro- and nanobeads, without using any additive. The particle size can be tuned by adjusting the concentration of the monomers and initiators.^24−26^ Moreover, the use of different polarity aprotic porogens can significantly alter the size of the MIP particles.^26^

Advantageous electrochemical activity, biocompatibility, rich surface chemistry, and strong resistance to biofouling made carbon nanomaterials helpful in building electrochemical chemosensors for biocompounds.^27^ Combining these nanomaterials, including carbon nanotubes, graphene, carbon dots, and nanodiamonds, with other materials to yield composites results in chemosensors revealing mechanical stability, high conductivity, and efficient signal transduction,^28−30^ thus improving their sensitivity and detectability.^31^ In particular, carbon nanotubes, a cylindrical seamless carbon material with a well-ordered arrangement of sp^2^-hybridized carbon atoms linked via π bonds, have extensively been exploited in biosensors as an attractive scaffold material.^27,28^

Herein, we devised a new MIP nanohybrid. To this end, first, we prepared DUL-imprinted MIP nanoparticles (nanoMIPs). Next, we sedimented those nanoMIPs together with single-walled carbon nanotubes (SWCNTs) on the electrode. Then, we potentiodynamically polymerized tyramine and simultaneously deposited a layer of the thus obtained polytyramine on this electrode. Due to the SWCNTs and polytyramine presence, the recognition unit of the MIP chemosensor thus fabricated resembled a network. NanoMIPs reveal a high ability to bind DUL. SWCNTs served as “electrical bridges” assisting in electron transfer between nanoMIPs and the electrode, and polytyramine bound these NPs. The nanoMIPs chemosensor selectivity to DUL appeared higher than to common interferences. Moreover, its sensitivity, durability, and determination repeatability were high. The chemosensor has been successfully used for DUL determination in human plasma. HPLC-UV determinations confirmed the practical usefulness of the chemosensor.

## 2 Experimental Section

### 2.1 Materials

DUL was synthesized in the Chemistry Department of the Łukasiewicz Research Network-Pharmaceutical Research Institute (presently Łukasiewicz Research Network-Institute of Industrial Chemistry), Warsaw, Poland. 2,2′-Azobis(2-methylpropionitrile) (AIBN), ethylene glycol dimethyl acrylate (EDGMA), methacrylic acid (MAA), acrylamide (AA), 2-hydroxypropyl methacrylamide (HPMA), *N,N’*-methylenebis(acrylamide) (BIS), tyramine, and solvents were from Sigma-Aldrich. SWCNTs (outer diameter < 2 nm, length 1–5 μm) were from SES Research (Houston, TX, USA). Solutions were prepared using deionized Milli-Q water (18.2 MΩ cm) from Merck Millipore.

### 2.2 Instrumentation

Section S1 (Supporting Information) describes the instruments applied.

### 2.3 Techniques and Procedures

#### 2.3.1 DUL-Imprinted nanoMIPs Synthesis via Precipitation Polymerization

For the nanoMIPs preparation, a mixture of DUL (0.1 mmol), MAA (0.5 mmol), EGDMA (2 mmol), and AIBN (0.02 mmol) was prepared (Figure 1). A 5-fold molar excess of the MAA functional monomer to the DUL template was used to increase the number of imprinted cavities in the resulting nanoMIPs. All of the components were dissolved in a 15 mL sample of anhydrous chloroform in a glass vial fitted with an airtight septum. Afterward, the resulting solution was deaerated by purging nitrogen for 15 min on ice. Polymerization was performed overnight at 65 °C in an oil bath. After polymerization, nanoMIPs were collected by centrifugation.

Non-imprinted polymer NPs, nanoNIPs, were synthesized similarly but without the DUL template.

#### 2.3.2 DUL Template Extracting from nanoMIPs

The DUL template was removed from the nanoMIPs by batch extraction using an (acetic acid)–methanol (1:9, *v*/*v*) mixture and then an (acetic acid)–ethanol mixture (1:9, *v*/*v*). Next, the nanoMIPs were twice washed with ethanol, followed by one round of methanol until no HPLC template peak was detected in the extracting solution.^32^ Subsequently, nanoMIPs were vacuum-dried overnight.

#### 2.3.3 NanoMIPs and nanoNIPs Immobilization on Electrodes for DUL Determination

NanoMIPs or nanoNIPs, with SWCNTs, were immobilized on the gold disk electrode by potentiodynamic electropolymerization of tyramine. They were integrated with the resulting thin polytyramine film via matrix entrapment.^33,34^

Toward that, SWCNTs (10 mg) were first dispersed in 10 mM tyramine in 25 mM H_2_SO_4_ for ∼150 min using an ultrasonic homogenizer. Following the so-called “one-pot” synthesis, the nanoMIPs (0.5 mg) were added to this dispersion (0.5 mL), and then it was ultrasonicated for 5 min. Next, this dispersion was sedimented for ∼75 min, followed by tyramine electropolymerization, where the potential was cycled five times between 0 and 1.50 V vs a Ag quasi-reference electrode at 50 mV s^–1^. Afterward, the electrode was rinsed with deionized water to remove any residual tyramine monomer, free SWCNTs, and nanoMIPs from the deposited composite film. Before further examination, the film was air dried.

#### 2.3.4 Human Plasma Sample Preparation for Determining DUL

A 1.0 mg/mL DUL stock solution was prepared by dissolving a weighed DUL portion in methanol. Working solutions were then prepared by this solution appropriately diluting with 50% methanol. Human plasma samples (with citrate as the anticoagulant) were spiked with the appropriate DUL working solution at a volume ratio of 20:1. Thus, the DUL concentration in plasma samples ranged from 33.6 to 840.6 nM (Table 1). Each sample was split into two parts for DUL determination with an electrochemical chemosensor featuring the nanoMIPs-SWCNTs@polytyramine film signal transduction unit and HPLC-UV.

**Table 1:** Comparison of Methods of DUL Determination in Human Plasma and a Test Solution Using HPLC-UV and the MIP-DUL Chemosensor

sample no.	HPLC-UV-determined DUL concentration in the test solution (nM)	HPLC-UV-determined DUL concentration in human plasma (nM)^a^	recovery (%)	known DUL concentration in the test solution (nM)	MIP-chemosensor-determined DUL concentration in the test solution (nM)	recovery (%)
1	33.6	33.2	98.8	3.3	4.0 (±1.25)	121.2 (±39.0)
2	100.8	100.5	99.7	10.0	10.6 (±2.47)	106.0 (±24.7)
3	336.2	332.2	98.8	33.6	30.2 (±8.75)	89.8 (±26.0)
4	571.6	577.0	100.9	57.1	59.5 (±12.8)	104.2 (±22.5)
5	840.6	802.6	95.4	84.0	89.4 (±11.0)	106.4 (±13.1)

a Arithmetic average (*n* = 6).

#### 2.3.5 DUL Determination in Human Plasma Using a nanoMIPs-SWCNT@polytyramine Film-Coated Electrode

The DUL-spiked human plasma samples of known DUL concentrations were thawed in air and then 10 times diluted with PBS (pH = 7.2). These samples were made to 10 mM in the K_3_[Fe(CN)_6_] and K_4_[Fe(CN)_6_] redox probes. A 1 mL sample of the DUL-spiked human plasma solution with this probe was consecutively placed in the electrochemical minivessel. Then, the nanoMIPs-SWCNT@polytyramine film-coated electrode was immersed in these solutions, and the normalized DPV peak current, *I*_DPV_, values were measured for DUL of known concentrations. The resulting changes in the *I*_DPV_ detection signal served for constructing the calibration plot for DUL.

#### 2.3.6 DUL Determination in Human Plasma Using HPLC-UV

DUL was also determined in human plasma using HPLC-UV to confirm the DUL chemosensing, complying with the OECD Principles of Good Laboratory Practice (GLP). First, the samples were extracted with *tert*-butyl methyl ether. Then, sample components were determined using HPLC-UV on a Symmetry C18 150 × 3.0 mm, 3.5 μm column (Waters, USA) at 25 (±2) °C. A 10 mM ammonium formate and acetonitrile (62.5:37.5, *v*/*v*) mixture served as the mobile phase. For DUL determination, 230 nm UV light was applied. Fluoxetine hydrochloride served as the internal standard. The complete analysis run time was 12 min.

#### 2.3.7 Other Procedures

Details of the computer simulations of polymer nanoparticle immobilization on Au-layered glass slides for SEM imaging are described in Sections S2 and S3 in the Supporting Information.

## 3 Results and Discussion

The present research aimed at devising nanoMIPs for electrochemical sensing of DUL in human plasma. First, the most appropriate functional and cross-linking monomers were selected by computer modeling. These monomers were used at different ratios to find nanoMIPs with the highest affinity to the target DUL analyte. After exhaustive characterization, the most promising nanoMIPs were immobilized on gold electrodes. The affinity, cross-reactivity, repeatability, and reproducibility in buffered solution samples were first investigated, followed by DUL determination in human plasma samples to validate the developed chemosensing system.

### 3.1 Functional and Cross-Linking Monomers Impact on the Prepolymerization Complex Stability

The structures of eight prepolymerization complexes of components with various molar ratios were simulated computationally. Selection of the complex composition was inspired by results for the nanoMIPs chemosensor based on MAA and EGDMA^35^ and (or) on structural similarity of components’ fragments. Consequently, MAA and three other functional monomers, vis., 4-VP, AA, and HPMA, and two cross-linking monomers, vis., EGDMA and BIS, were considered. The most stable prepolymerization complex, **S1c**, is formed at a MAA-to-EGDMA molar ratio of 5:20, as shown in Figure S1. The resulting values of the Gibbs free energy change, Δ*G*_C_, are presented in Section S4 and Table S2 in the Supporting Information for all systems tested.

### 3.2 nanoMIPs Synthesis via Precipitation Polymerization and Their Characterization

Once the functional monomer, cross-linking monomer, and their optimum ratios for stable prepolymerization complex formation were selected through computational modeling, nanoMIPs and the corresponding nanoNIPs were synthesized and characterized.

#### 3.2.1 nanoMIPs and nanoNIPs Size and Zeta Potential Measurements

**Figure 2:**
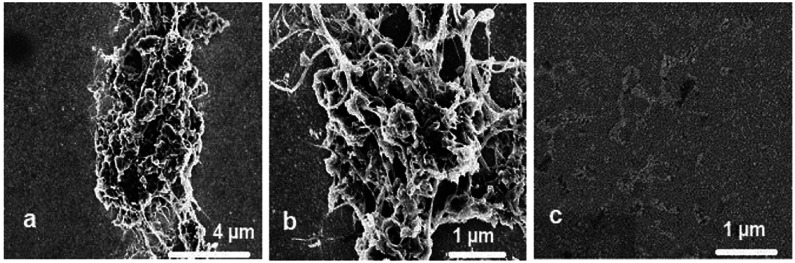
SEM images at different magnifications of (a and b) nanoMIPs embedded in the SWCNTs–polytyramine film and (c) SWCNTs-polytyramine film without nanoMIP. Films were deposited on Au-layered glass slide electrodes.

Dynamic light scattering (DLS) determined the hydrodynamic size and zeta potential of the nanoMIPs and nanoNIPs. For DLS measurements, 1 mg/mL nanoMIPs and nanoNIPs samples were ultrasonicated in deionized water. The average sizes of the nanoMIPs and nanoNIPs were determined to be 157 (±14) and 529 (±18) nm, respectively. The zeta potentials for the nanoMIPs and nanoNIPs were −9.6 and −46.3 mV, respectively. It revealed the net surface charge and, hence, long-term stability of the nanoparticles. Since the nanoMIPs negative zeta potential is small, these nanoMIPs rapidly agglomerate (Figure 2b). In contrast, nanoNIPs are stable with respect to aggregation because of the much larger negative zeta potential and, hence, stronger electrostatic repulsions between nanoMIPs.

During nanoMIP formation, one DUL molecule interacts with five MAA molecules. Then, after DUL extraction, unbound MAA is washed off. Because this process is stoichiometrically controlled, a smaller number of MAA molecules could be incorporated into the polymer for nanoMIPs compared to polymerization, leading to nanoNIPs. DUL is absent during the nanoNIPs synthesis. Thus, possibly, several molecules of MAA, its dimer, and EGDMA mutually interact, leading to a higher MAA content in the nanoNIPs. As the MAA can only be charged in the polymer formed, a less negative surface charge on nanoMIPs is generated. However, during the nanoNIPs synthesis, several molecules of MAA, its dimer, and EGDMA may interact with each other. Due to this higher MAA concentration in nanoNIPs, the overall surface charge on nanoNIPs is more negative than on that on nanoMIPs.

#### 3.2.2 SEM Imaging of nanoMIPs and nanoNIPs

After nanoMIPs-SWCNTs@polytyramine film deposition, the morphological and structural film changes were monitored by SEM imaging. That confirmed formation of a web-like structure where nanoMIPs were encapsulated in the SWCNTs-polytyramine film (Figure 2a and 2b), unlike the SWCNT-polytyramine film (Figure 2c).

#### 3.2.3 Electrochemical Characterization of Electrode-Immobilized nanoMIPs

NanoMIPs should be placed as close as possible to the electrode surface so that after binding the DUL analyte, the transducer, here, the electrode, generates a well-pronounced analytical signal. This can be achieved with a cross-linking monomer that joins the molecules in prepolymerization complexes and binds the polymer nanoparticles to the transducer surface during electropolymerization. For that, we used tyramine which, upon electropolymerization, forms a thin polymer film. This film is widely applied for tissue repair and drug release.^36^

**Figure 3:**
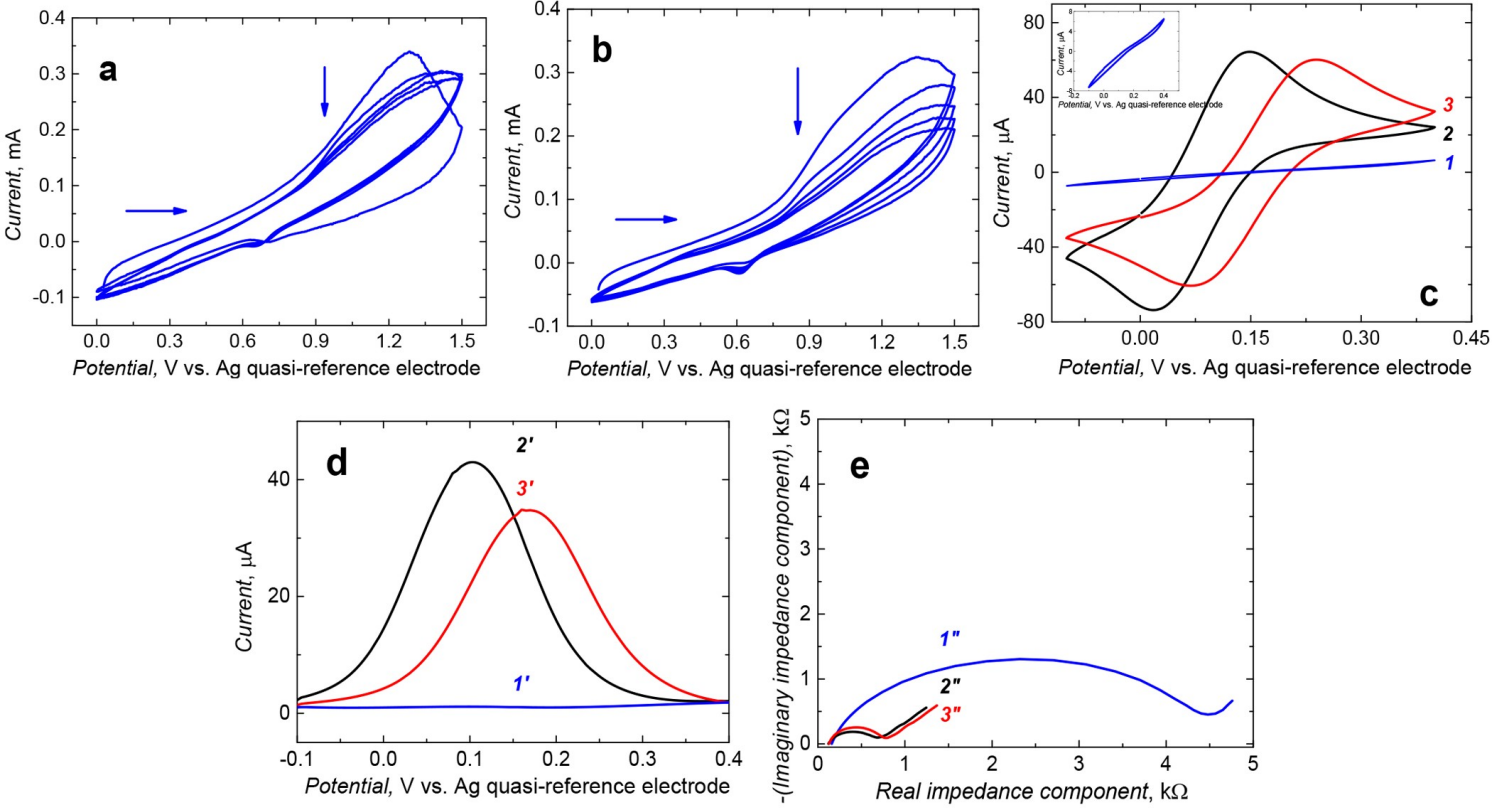
Multicyclic potentiodynamic curve for a mixture of 10 mM tyramine in 25 mM H_2_SO_4_ and SWCNTs on a 2 mm diameter Au-disk electrode at a 50 mV s^–1^ potential scan rate in the (a) absence and (b) presence of nanoMIPs sedimented on the electrode surface for 75 min. (c) CV and (d) DPV voltammograms and (e) EIS curves at 0.15 V vs Ag quasi-reference electrode for 10 mM K_3_[Fe(CN)_6_] and 10 mM K_4_[Fe(CN)_6_] in 0.1 M PBS (pH = 7.2) recorded on the Au disk electrode coated with a film of (*1*, *1’*, and *1′′*) nanoMIPs@polytyramine, (*2*, *2’*, and *2′′*) SWCNTs@polytyramine, and (*3*, *3′*, and *3′′*) nanoMIPs-SWCNTs@polytyramine.

NanoMIPs and SWCNTs were immobilized in a polytyramine film using five potentiodynamic cycles (Figure 3). In the resulting multicyclic potentiodynamic curve (Figure 3b), two anodic peaks at ∼0.99 and ∼1.34 V and one cathodic peak at ∼0.61 V vs the Ag quasi-reference electrode are present. Anodic currents decreased in consecutive cycles, indicating that electrode coating with a nonconducting polymer film was thicker after each cycle. This behavior resembles the SWCNTs@polytyramine film potentiodynamic deposition in the nanoMIPs absence (Figure 3a), confirming polytyramine film deposition. The SWCNTs’ presence in the nanoMIPs@polytyramine film increased the currents, demonstrating that SWCNTs play a vital role in electron transfer. The slope of the calibration plot for DUL at the nanoMIP-SWCNT@polytyramine film-coated electrode was ∼4 times higher than that at the nanoMIPs@polytyramine film-coated electrode (Figure S2, Supporting Information).

Expectedly, the CV (Figure 3c) and DPV (Figure 3d) peaks of the K_4_[Fe(CN)_6_]/K_3_[Fe(CN)_6_] probe in PBS (pH = 7.2) at the nanoMIP-SWCNT@polytyramine film-coated electrode were lower than those at of the SWCNTs@polytyramine film-coated electrode. These peaks were exploited to confirm the successful immobilization of nanoMIPs in the SWCNTs@polytyramine film (Figure 3c–3e), that is, both CV and DPV peaks for the nanoMIPs-SWCNTs@polytyramine film-coated electrode (curve *3* in Figure 3c and curve *3′* in Figure 3d, respectively) were significantly smaller than those for the SWCNTs@polytyramine film-coated electrode (curve *2* in Figure 3c and curve *2’* in Figure 3d, respectively). Moreover, the semicircle diameter corresponding to the charge transfer resistance, *R*_ct_, in the Nyquist plot for the nanoMIPs-SWCNTs@polytyramine film-coated electrode was larger than that for the SWCNTs@polytyramine film-coated electrode (curves *3′′* and *2′′*, respectively, in Figure 3e), thus manifesting more extensive blocking of the former electrode.

#### 3.2.4 DPV Determination of the DUL Using the nanoMIPs-SWCNTs@polytyramine Film-Coated Electrode

**Figure 4:**
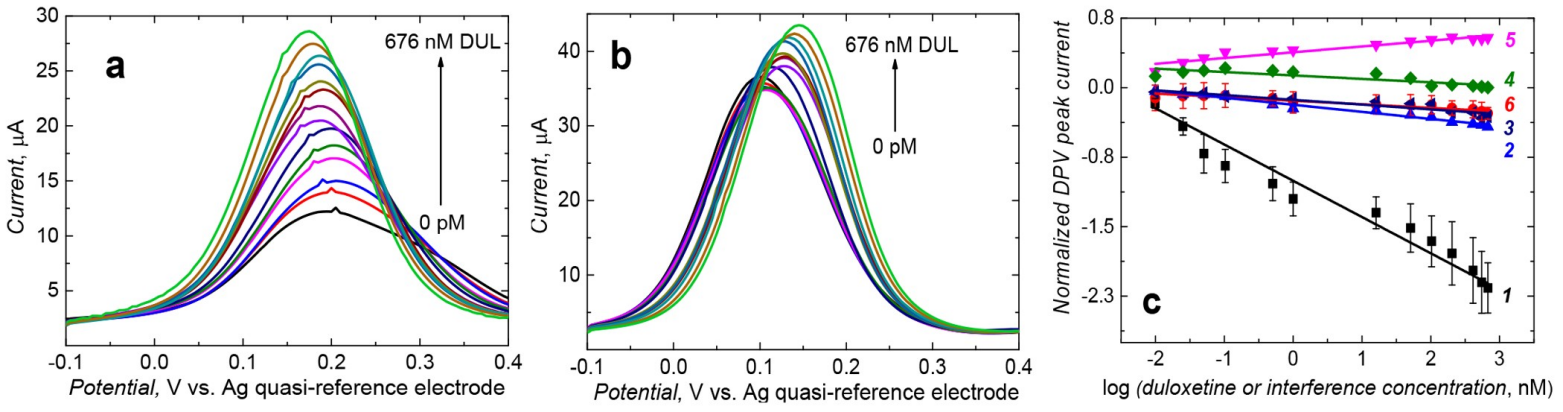
DPV peaks recorded at 2 mm diameter Au disk electrodes coated with the SWCNT–polytyramine films containing (a) nanoMIPs and (b) nanoNIPs in the presence of DUL of different concentrations indicated in the curves in 10 mM K_3_[Fe(CN)_6_] and 10 mM K_4_[Fe(CN)_6_] in 0.1 M PBS (pH = 7.2). (c) Calibration plots of DPV-normalized peaks constructed using electrodes coated with (curves *1*–*5*) nanoMIPs-SWCNTs@polytyramine and (curve *6*) nanoNIPs-SWCNTs@polytyramine films. Curves *1*, *2*, *3*, *4*, and *5* are respective calibration plots for DUL, creatinine, urea, glucose, and cholesterol.

NanoMIP-SWCNT@polytyramine film-coated Au-disk electrodes were used for DUL determination. For that, the DPV (Figure 4a and 4b) and EIS (Figure S3, Supporting Information) responses to the K_4_[Fe(CN)_6_]/K_3_[Fe(CN)_6_] probe were measured. The normalized DPV peak (*I*_DPV,0_ – *I*_DPV,s_)/*I*_DPV,0_, where *I*_DPV,0_ and *I*_DPV,s_ stand for the initial and actual DPV current peak, linearly depended on the logarithm of the DUL concentration (Figure 4c). The linear dynamic concentration range extended from 10 pM to 676 nM DUL, obeying the regression equation (*I*_DPV,0_ – *I*_DPV,s_)/*I*_DPV,0_ = −0.39 (±0.021)/log [nM] × log *c*_DUL_ [nM] – 1.00 (±0.038) (Figure 4c, curve *1*), where *c*_DUL_ is the DUL concentration. The sensitivity and correlation coefficient were −0.39 (±0.021)/log [nM], and 0.96, respectively. At the signal-to-noise ratio, S/N = 3, the chemosensor’s LOD was 1.6 pM DUL, being adequately low for the DUL determination in body fluids.

Moreover, the DPV signal for the nanoNIPs-SWCNTs@polytyramine film-coated electrode did not change much with increasing DUL concentration, thus indirectly confirming successful imprinting. This signal is described by the semilogarithmic regression equation (*I*_DPV,0_ – *I*_DPV,s_)/*I*_DPV,0_ = −0.03 (±0.003)/log [nM] × log *c*_DUL_ [nM] – 0.14 (±0.007) (Figure 4c, curve *6*) for the concentration range of 10 pM to 923 μM DUL. The sensitivity and correlation coefficient were −0.03 (±0.003)/log [nM] and 0.89, respectively. The apparent imprinting factor was estimated from the ratio of the slopes of the DUL calibration plots for the nanoMIPs-SWCNTs@polytyramine and nanoNIPs-SWCNTs@polytyramine film-coated electrodes. Advantageously, it was very high, equaling *IF* = 13.0.

After the DPV curves were recorded, the EIS spectra (Section S5, Supporting Information) were recorded for the same solutions to gain insight into the mechanistic aspects of the chemosensor response. Moreover, analytical parameters, including the fabricated chemosensor’s linear dynamic concentration range and limit of detection, were compared to those already reported in the literature (Table S1). Apparently, the herein fabricated chemosensor outmatches all of those previously reported.

### 3.3 Cross-Reactivity Study

Although the chemosensor detectability of the target DUL analyte was adequate, it was necessary to test the chemosensor for selectivity against common interferences encountered in human plasma (Figure 4c).

Cross-reactivity experiments were performed with four common interferences, including urea, glucose, creatinine, and cholesterol (Figure S4, Supporting Information), at the same concentration order as DUL to determine the selectivity coefficient (α) values (Figure 4c). Ratios of the slopes of the DUL calibration plots to those of the interference were calculated. Advantageously, the chemosensor was not responsive to cholesterol. In summary, integrating the nanoMIPs-SWCNTs@polytyramine film with the electrode formed a complete chemosensor that was appreciably selective to common interferences.

### 3.4 nanoMIPs-SWCNTs@polytyramine Stability and Reusability

Two essential criteria required for any sensing device, besides sensitivity and selectivity, are the stability and reusability. Our chemosensor was stable for at least 2 months with only a 4.0% signal decay (Figure S5, Supporting Information) and could be reused at least five times without significant DUL sensing ability loss. DUL was extracted with methanol for ∼30 min after each determination for chemosensor reuse.

### 3.5 Computer Calculations

#### 3.5.1 Computational Modeling of the nanoMIP Cavity as Well as Simulating Analyte and Interferences Sorption in This Cavity

**Figure 5:**
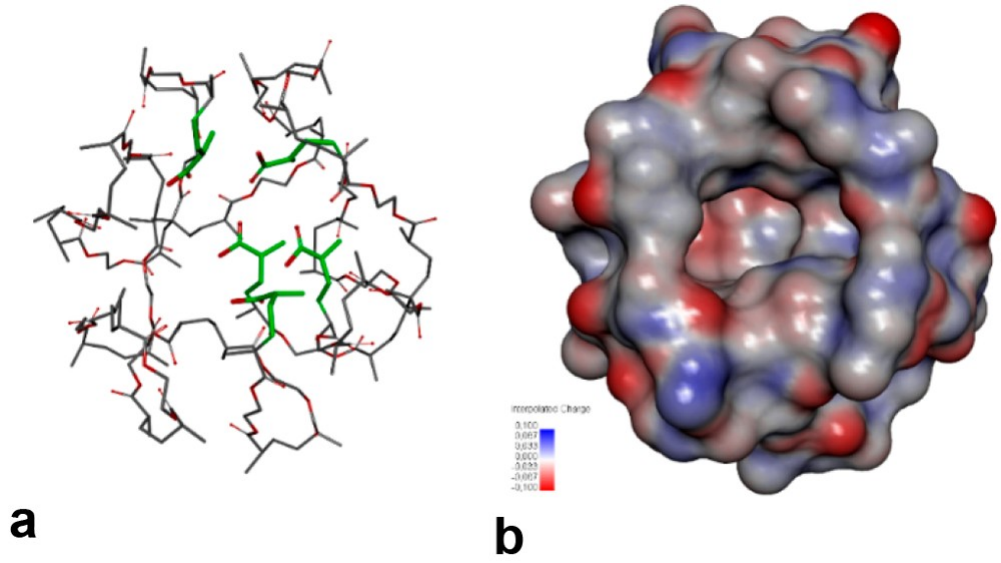
Computationally modeled structure of the molecular cavity imprinted in DUL-nanoMIP: (a) skeleton model; (b) surface distribution of the molecular electrostatic potential (MEP) colored according to the interpolated (blue) positive and (red) negative charge.

The model of the cavity in the polymer matrix was set up based on the **S1c** complex (Section 2, Supporting Information). Figure 5a and 5b presents the respective polymer cavity’s skeleton model and the molecular electrostatic potential (MEP) distribution on the cavity surface. The negative potential areas predominate at the back of the cavity in the proximity of the oxygen atoms of the carbonyls and carboxyls, while the positive potential areas are located close to the cavity edge. The positive potential areas are encountered near the hydrogen atoms of the hydroxyls.

The computed values of the Gibbs free energy change resulting from nanoMIPs binding of the analyte or interferences (Δ*G*_bind_) are presented in Table S3 (Supporting Information). The nanoMIPs strongest interactions are predicted for DUL, corresponding well to a high experimental imprinting factor. The Gibbs binding free energy changes computed for the creatinine, urea, glucose, and cholesterol interferences are much lower. Hence, those should not interfere with the DUL determination. The experimental selectivity coefficient (α) calculated from the DPV experiments correlates with the calculated Δ*G*_bind_, confirming the computational selectivity predictions (Table S3, Supporting Information).

#### 3.5.2 Modeling of nanoMIPs Cavity Interactions with Molecules of the DUL Analyte and Molecules of Interferences

The recognition properties of the MIP cavities are defined by the difference in the strength of their interactions with the analyte and interferences. The main structural features responsible for the nanoMIPs selectivity are described (Section S6, Supporting Information) based on the DUL analyte and the glucose, urea, creatinine, and cholesterol interferences sorption. The interference molecules can penetrate the cavity (Figure S6 and S7, Supporting Information), but having different structures, shapes, and sizes, they cannot interact as strongly as DUL. Besides, glucose, urea, creatinine, and cholesterol molecules are neutral unlike the DUL molecule, which is positively charged on its amino group.

Moreover, creatinine can exist in two tautomeric forms, vis., as the 2-imino-1-methyl-2-imidazolidine-4-one imino tautomer and the 2-amino-1-methyl-2-imidazoline-4-one amino tautomer (Figure S4 in Supplementary Information).^37^ Therefore, both forms were tested herein. Both tautomers are located in the cavity center, and water molecules help keep them inside via interactions with −C=O or −NCH3 moieties (Figure S7c in Supplementary Information). These interactions are not responsible for molecular recognition. The Δ*G*_bind_ values indicate that the nanoMIP cavity binds the amino tautomer stronger than the imino tautomer (Δ*G*_bind_ = -55.54 kJ/mol). The amine group of (2-amino-1-methyl-2-imidazoline-4-one) is oriented to the cavity wall, but the imine group of (2-imino-1-methyl-2-imidazolidine-4-one) is directed down outside the cavity (Figure S7a in Supplementary Information).

In summary, the MAA and EGDMA moieties’ oxygen atoms’ interactions with the amino moiety and the thiophene ring of the DUL analyte can play a crucial role in the molecular recognition ability of the DUL-nanoMIP matrix. The DUL-nanoMIP selectivity to DUL metabolites is described in Section S7 and Figures S8 and S9 (Supporting Information).

### 3.6 DUL Determination in Human Plasma Using the nanoMIPs-SWCNT@polytyramine Chemosensor

The practical usability of a newly fabricated chemosensor should be evaluated using real samples to estimate the effect of the matrix. Accordingly, the present chemosensor performance was investigated for DUL-spiked human plasma samples (Table 1). These samples, anticoagulated with citrate, were than 10 times diluted with PBS (pH = 7.2). Significantly, the chemosensor successfully determined DUL in the plasma samples using DPV.

## 4 Conclusions

We developed a method for sensitive and selective duloxetine (DUL) determination in human plasma. To this end, we devised, fabricated, and tested a new nanoMIPs-based electrochemical chemosensor. DUL-imprinted nanoMIPs were immobilized in a polytyramine film deposited by electropolymerization on SWCNTs sedimented on a transducer (electrode) surface in this chemosensor. The most appropriate functional and cross-linking monomers to obtain nanoMIPs with a high affinity to the target DUL analyte were selected by computational simulations. The recognizing properties and stability of the nanoMIPs were high. The linear dynamic concentration range extends from 10 pM to 676 nM DUL, outmatching all previously reported ranges by several orders of magnitude. Both the DPV and the EIS chemosensors engineered herein are suitable for determining DUL at LODs of 1.6 and 2.0 pM, respectively, which are well below the limit of 33 nM adopted in clinical practice. Yet, the DPV chemosensor outperformed the EIS chemosensor in all aspects of DUL chemosensing. The DPV peaks for the DUL analyte in the presence of interferences, commonly encountered in human plasma, were at least five times smaller than those for this analyte in PBS (pH = 7.2). The chemosensor durability (at least 2 months), reusability (at least five times), and repeatability are high. Hence, the chemosensor is beneficial for clinical analysis due to the possibility of DUL sensing in human plasma.

## Supporting Information Available

Instrumentation, computer simulations, polymer nanoparticles immobilizing on glass slides for SEM imaging, functional and cross-linking monomers influence on prepolymerization complex stability, table with the composition of prepolymerization complexes and their Gibbs free energy changes, stability of the chemosensor, DUL determination with EIS, structural formulas of the interferents, calculated changes of the Gibbs free energy (Δ*G*_bind_) due to nanoMIP binding of the DUL analyte and selected interferences, modeling of nanoMIPs cavity interactions with molecules of the DUL analyte and interferences, DUL–nanoMIP selectivity to DUL metabolite (PDF)
