# Supplementary material for: Polytyramine Film-Coated Single-Walled Carbon Nanotube Electrochemical Chemosensor with Molecularly Imprinted Polymer Nanoparticles for Duloxetine-Selective Determination in Human Plasma
Source: ACS Sens. 2022 May 12;7(7):1829–36. doi: 10.1021/acssensors.2c00124 (PMC9315955; doi:10.1021/acssensors.2c00124)
Supplement: Supplementary file 1 — se2c00124_si_001.pdf [file se2c00124_si_001.pdf]

Supplementary Information

for

Polytyramine Film-Coated Single-Walled Carbon Nanotube Electrochemical Chemosensor with Molecularly Imprinted Polymer Nanoparticles for Duloxetine-Selective Determination in Human Plasma

Jyoti,[a] Teresa Żołek,[b] Dorota Maciejewska,[b] Edyta Gilant,[c] Elzbieta Gniazdowska,[c] Andrzej Kutner,[d] Krzysztof R. Noworyta,[a,*] and Wlodzimierz Kutner [a,e,*]

[a] *Institute of Physical Chemistry, Polish Academy of Sciences, Kasprzaka 44/52, 01-224 Warsaw, Poland*

[b] *Department of Organic Chemistry, Faculty of Pharmacy, Medical University of Warsaw, 1 Stefana Banacha, 02-097, Warsaw, Poland*

[c] *Łukasiewicz Research Network – Industrial Chemistry Institute, Rydygiera 8, 01-793, Warsaw, Poland*

[d] *Department of Bioanalysis and Drug Analysis, Faculty of Pharmacy, Medical University of Warsaw, Banacha 1, 02-097 Warsaw, Poland*

[e] *Faculty of Mathematics and Natural Sciences. School of Sciences, Institute of Chemical Sciences, Cardinal Stefan Wyszynski University in Warsaw, Wóycickiego 1/3, 01-815 Warsaw, Poland*

Corresponding author email addresses: knoworyta@ichf.edu.pl and wkutner@ichf.edu.pl

Table S1. Comparison of parameters of chemosensors used for DUL determination.

Chemosensor composition or determination principle	DUL linear dynamic concentration range	DUL limit of detection (LOD)	Detection method	Ref.
Ion-selective membrane electrode on a Cu wire	10 µM – 10 mM	10 µM	Potentiometric	1
Ion selective membrane electrode	10 µM – 10 mM	6.31 µM	Potentiometric	2
Carbon paste electrode	0.2 – 5.0 µM<br>0.07 – 1.0 µM	6.0 nM (LSV)<br>21 nM (SWV)	LSV, SWV	3
Multi-wall carbon nanotubes modified carbon paste electrode	3.0 µM – 0.2 mM	0.4 µM	CV, SWV	4
Screen-printed disposable electrode	1.0 µM – 10 mM	0.5 µM	Potentiometric	5
-	59 nM – 1.19 µM	8.9 nM	Spectrofluorimetric	6

S1. Instrumentation

An SP-300 potentiostat/galvanostat computerized electrochemical system of Bio-Logic, SAS, controlled by EC-Lab v10.37 software of the same manufacturer, was used for electrochemical measurements. These measurements were performed using a three-electrode one-compartment V-shaped glass electrochemical minicell. A PEEK shrouded 2-mm diameter Au disk and Ag and Pt wires, respectively, served as the working, quasi-reference, and counter electrode.

All DPV and EIS measurements were performed using the electrochemical minivessel described above and the phosphate-buffered saline, PBS, (pH=7.2) solution of the 10 mM $K_3[Fe(CN)_6]$ and 10 mM $K_4[Fe(CN)_6]$ redox probe. In the DPV measurements, the potential was scanned from -0.10 to 0.40 V vs. Ag/AgCl with the potential step of 5 mV and the applied amplitude of 50-ms pulses of 25 mV. In the EIS experiments, an ac excitation signal of frequency in the range of 1 MHz to 100 mHz and a 10-mV sinusoidal amplitude were used at the applied potential of 0.15 V vs. Ag/AgCl. The electrochemical system was approximated with a modified Randles-Erschler equivalent circuit, $R_s+CPE/(R_{ct}+W_o)$, with Z-View software (Scriber Associates, Inc.). The R_s, R_{ct}, CPE, and W_o symbols represent the solution resistance, charge transfer resistance, constant phase element, and Warburg impedance.

The DLS measurements were performed using a Zetasizer Nano series (Malvern Instruments, Ltd).

The MIP nanoparticles (nanoMIPs), immobilized in a polytyramine film coating SWCNTs deposited on electrodes, were imaged with scanning electron microscopy (SEM) using a Nova NanoSEM 450 microscope of the FEI Nova. For imaging, a dedicated three-electrode one-compartment Teflon electrochemical minivessel was used to immobilize the nanoMIPs in the solution mixture of SWCNTs and polytyramine films deposited on the Au-layered glass slide working electrodes. An inner diameter of the O-ring used limited the active film diameter to ~2 mm. An Ag wire and a Pt wire served as the reference and counter electrode, respectively. The vessel set was designed and fabricated in the IPC PAS machine shop.

The DUL extraction from MIP was confirmed with HPLC using an Agilent Infinity 1290 LC system equipped with the DAD detector and the Thermo Hypercyl GOLD C18 (100 × 2.1 mm, 1.9 μm) column of Thermo Fisher Scientific.

All measurements were performed at room temperature, 20 (±1) °C.

S2. Computer simulations

The MIPs' structures and interactions were simulated using Discovery Studio 2017R2 BIOVIA software.[7] Molecular structures of all compounds, namely, DUL, MAA, 4-VP, AA, HPMA, EGDMA, and BIS in the chloroform solvent environment, were optimized at the density functional theory (DFT) level using the B3LYP/6-311+G(d,p) hybrid functional implemented in Gaussian 16 program.[8] Using Breneman's model (Breneman and Wiberg, 1990), we assigned partial atomic charges to the atoms, thus reproducing the molecular electrostatic potential (MEP). The pre-polymerization complexes, the cavity in nanoMIP, the polymerization, and the sorption processes were simulated to interpret the physicochemical properties of the tested systems. The computations were carried out using a CHARMm force field.[9] The systems' energy was initially optimized at the molecular mechanics (MM) level using 1000 steepest descents and 10,000 conjugate gradient steps to remove ineffective contacts.

Further, the molecular dynamics (MD) simulations were carried out for 5 ns. The MD protocol contained a step of heating from 0 to 339 K, performed for 100 ps with time steps of 1 fs. Before the production stage, the system was isothermally equilibrated for 100 ps at 339 K. The Leapfrog Verlet integration and SHAKE algorithms were used for this simulation.[10] Both temperature and pressure were controlled using the Berendsen method. The coordinates were

recorded every 10 ps. Trajectory structures for analysis were saved at 1 ps. The energies and the structures used in all analyzes were from the trajectory file data generated from the last 2 ns MD simulations. The MM and MD computations were accomplished using an appropriate solvent and an explicit solvation model. The steps of polymerization in chloroform (in accord with the experiment) were simulated by adding 550 chloroform molecules to the system and using PACKMOL software.[11] As for proceeding in aqueous solution at pH=7.4, the analyte and interferences sorption was analyzed within the TIP3P model[12] using a cubic box of water molecules extending up to 10 Å from any solute atom neutralized by chloride anions added.

MIP properties were determined using the structure of the pre-polymerization complex built of the DUL template molecule interacting with four different functional monomer molecules and two cross-linking monomer molecules. For that, determining the most appropriate molecular composition of the complex was an essential part of the studies preceding the nanoMIP synthesis. The models of selected pre-polymerization DUL complexes with four different functional monomers and two cross-linking monomers were built. First, the complex systems of the DUL-to-MAA stoichiometric ratio of 1 : 3, 1 : 4, 1 : 5, and 1 : 6 were analyzed. The systems were generated by surrounding a DUL molecule with twelve MAA molecules. Then, after the energy optimization, the appropriate numbers of MAA molecules, i.e., 3, 4, 5, or 6, were selected to create the center of the pre-polymerization complexes. The main criteria for the selection involved the strongest interactions of DUL with the monomers of the hydrogen bond and π-π interactions. Then structures of these complexes were optimized again, and an auxiliary restraining potential was applied to the monomers in subsequent computations to preserve the internal geometry. A force constant of 41.8 kJ/mol $Å^2$ was sufficient for the calculations. Next, twenty molecules of the cross-linking monomer were added to the systems, and that way, complete starting structures of the pre-polymerization complexes were created. With the use of MM and MD, four models of the pre-polymerization complexes of DUL with MAA and EGDMA were constructed, named **S1a** (MAA : EGDMA, 3 : 20), **S1b** (MAA : EGDMA, 4 : 20), **S1c** (MAA : EGDMA, 5 : 20), **S1d** (MAA : EGDMA, 6 : 20). The Gibbs free energy changes accompanying the complexation were defined by intermolecular interactions between the template and functional or cross-linking monomer. The most appropriate stoichiometric ratio for the nanoMIP synthesis was chosen based on these calculations. In subsequent computations, three functional monomers (4-VP, AA, and HPMA) and two cross-linking monomers (EGDMA

and BIS) were considered by simulating four pre-polymerization complex systems at the 1 : 5 : 20 molar ratio, namely, **S2** (MAA : BIS, 5 : 20), **S3** (4-VP : EGDMA, 5 : 20), **S4** (AA : BIS, 5 : 20), and **S5** (HPMA : BIS, 5 : 20).

The final stage of structural modeling consisted of creating the MIP molecular cavity. That used the most stable pre-polymerization complex structure of the DUL: MAA : EGDMA ratio of 1 : 5 : 20, predicted for the complex synthesis. Single bonds between closely spaced carbon atoms of the C=C bonds of the MAA and EGDMA molecules were formed to mimic the polymerization. Then, the hydrogen atoms were added to the rest of the C=C bonds to remove the π-π interactions from the polymer matrix. The MD procedure was used to optimize the simulated system after calculating electrostatic potentials (the so-called ESP charges) for the proposed MIP matrix. Next, the template was removed from the resulting MIP, and the space vacated was proposed as the theoretical model of the sorption cavity in nanoMIP. The cavity model was used for analyzing analyte and interferences sorption in aqueous solutions. The DUL analyte and creatinine, urea, glucose, and cholesterol interferences were successively inserted in the cavity, and then the MD simulated their sorption. The constraints were set on heavy atoms of the cavity with the 418.68 kJ/mol $Å^2$ force constant for immobilizing the 3D cavity structure. The analytes were left freely, mimicking the sorption step. The changes of the Gibbs free energy (ΔG_{bind}) accompanying the nanoMIPs binding of the DUL and interference molecules were calculated using Equation S1.

$$\Delta G_{bind} = \Delta G_{system} - \Delta G_{cavity} - \Delta G_{DUL/interference} \quad \text{(Equation S1)}$$

In this equation, ΔG_{system} is the Gibbs free energy change attributed to the formation of a complex of the imprinted cavity with the analyte or interference molecule, ΔG_{cavity} is the Gibbs free energy of cavity formation, and $\Delta G_{DUL/interference}$ is the Gibbs free energy change due to the DUL or interference formation.

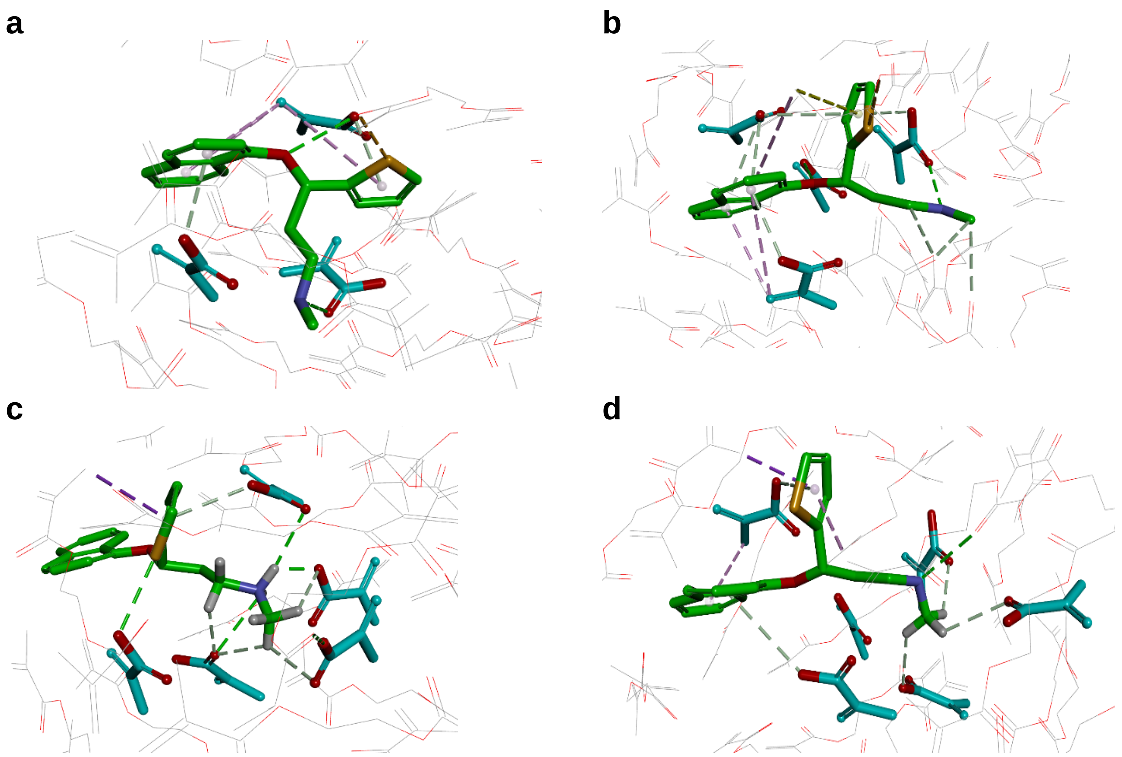

Figure S1. Simulated models of pre-polymerization complexes of DUL with MAA, surrounded by EGDMA molecules, at different molar ratios. (a) **S1a** (MAA : EGDMA, 3 : 20), (b) **S1b** (MAA : EGDMA, 4 : 20), (c) **S1c** (MAA : EGDMA, 5 : 20) and (d) **S1d** (MAA : EGDMA, 6 : 20). Significant intermolecular interactions are indicated with dashed lines.

S3. Polymer nanoparticles immobilizing on Au-layered glass slides for SEM imaging

The method of nanoMIPs immobilization described above was used for SEM imaging. To this end, an Au-layered glass slide was first washed with the "piranha" solution (H_2SO_4 : H_2O_2; 4 : 1, *v*/*v*) for 10 min, then rinsed with deionized water, and then carefully dried with a nitrogen stream. (*Warning. The "piranha" solution is hazardous if it comes in contact with skin or eye.*) This slide was then placed in the homemade Teflon cell with a 2-mm diameter O-ring. Then, the polymer NPs were suspended in the solution filling the cell for ~75 min for electropolymerization, followed by electropolymerization of tyramine with five potentiodynamic cycles. That led to the immobilization of the NPs in the polytyramine film deposited on SWCNTs.

S4. Functional and cross-linking monomers influence on pre-polymerization complex stability

Figure S1 in Supplementary Information shows spatial conformations of four complexes of DUL, MAA, and EGDMA at different DUL-to-MAA molar ratios. The more functional monomer molecules in the complex, the stronger are their interactions with the template and the higher the stability of the pre-polymerization complex. In complex **S1a** (1 : 3 : 20) (Figure S1a in Supplementary Information), MAA molecules generate two strong hydrogen bonds with DUL and only a few interactions of π-alkyl and π-donor types. However, more contacts were established in the **S1b** (1 : 4 : 20) and **S1c** (1 : 5 : 20) complexes. The strongest interactions are the hydrogen bonds formed by the carboxyl of MAA with the methylamine moiety (lengths of 1.96 to 2.68 Å) and the thiophene ring (length of 2.75 Å) of DUL. Besides, DUL is involved in new interactions of the π-donor type with other MAA molecules. If the number of functional monomer molecules is increased to six (complex **S1d**), they interact mutually. Hence, the template complexation is less efficient, and the stability of the pre-polymerization complex is lower. The EGDMA forms a network of interactions with all components of the complexes and affects these complexes' stability. BIS was the other cross-linking monomer selected. The **S2** complex of the DUL : MAA : BIS composition of the molar ratio of 1 : 5 : 20 was constructed and analyzed. The EGDMA replacement for BIS decreased the stability of the complex dramatically (Table S2 in Supplementary Information). The AA monomer replacement with HPMA in **S2** (see **S4** and **S5**) did not increase the stability of the complex systems. The AA, HPMA, and BIS amide groups interact with DUL ineffectively. Therefore, only EGDMA seems to be the cross-linking monomer suitable for the nanoMIPs. The last system simulated, **S3**, contained 4-VP (4-VP : EGDMA, 5 : 20). Even though the aromatic ring of 4-VP favorably interacts with aromatic moieties of DUL, the **S3** stability is much lower than that of **S1c**. To sum up, MAA and EGDMA at the molar ratio of 5 : 20 can be proposed as the functional and cross-linking monomers efficient in DUL imprinting in the polymer matrix.

Table S2. Compositions of mixtures for preparation of pre-polymerization complexes and the Gibbs free energy changes, ΔG_C ($\Delta G_C = \Delta G_{system} - \Delta G_{DUL} - n \cdot \Delta G_{FM} - 20 \cdot \Delta G_{CLM}$), for eight complex systems simulated.

Complex system*	ΔG_C kJ/mol
**S1a** (MAA : EGDMA, 3 : 20)	-313.95
**S1b** (MAA : EGDMA, 4 : 20)	-342.69
**S1c** (MAA : EGDMA, 5 : 20)	-343.81
**S1d** (MAA : EGDMA, 6 : 20)	-321.35
**S2** (MAA : BIS, 5 : 20)	-39.14
**S3** (4-VP : EGDMA, 5 : 20)	-248.68
**S4** (AA : BIS, 5 : 20)	-11.96
**S5** (HPMA : BIS, 5 : 20)	-13.23

*MAA – methacrylic acid, 4-VP – 4-vinylpyridine, AA – acrylamide, HPMA – *N*-(2-hydroxypropyl) methacrylamide, EGDMA – ethylene glycol dimethacrylate, BIS – *N,N′*-methylenebisacrylamide. The ΔG_{system}, ΔG_{DUL}, ΔG_{FM}, ΔG_{CLM}, and n symbols respectively stand for the Gibbs free energy change accompanying the formation of the system, DUL, functional and cross-linking monomers, and the number of functional monomer molecules in the complex.

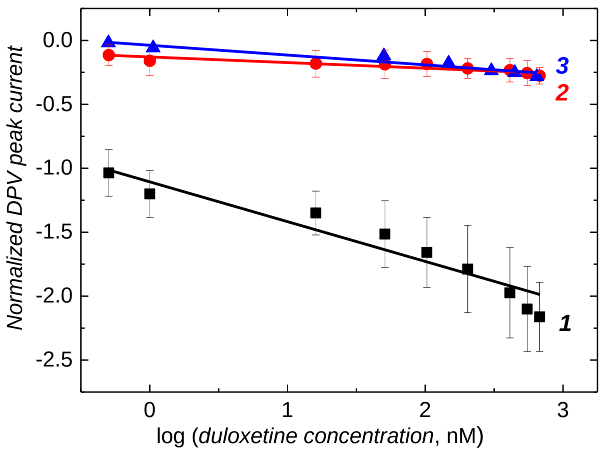

Figure S2. Calibration plots of normalized DPV peak current constructed using electrodes coated with the (curve *1*) nanoMIPs-SWCNTs@polytyramine, (curve *2*) nanoNIPs-SWCNTs@polytyramine, and (curve *3*) nanoMIPs@polytyramine film.

S5. DUL determining with EIS

In Nyquist plots, the semicircle diameter corresponds to the redox probe faradaic process's charge transfer resistance, R_{ct}. The semicircles were well pronounced for nanoMIPs and nanoNIPs (Figure S3a and S3b, Supplementary Information). A modified Randles-Ershler equivalent circuit was fitted to these spectra (Figure S3d, Supplementary Information). For nanoMIP-SWCNT@polytyramine, the R_{ct} linearly increased with the logarithm of DUL concentration (Figure S3 Supplementary Information), fulfilling the following semilogarithmic regression equation, $(R_{ct,0} - R_{ct,s})/R_{ct,0}$=0.10 (±0.003)/log [nM] × log (c_{DUL}, nM) + 0.29 (±0.007). The sensitivity and regression coefficient were 0.10 (±0.003)/log [nM] and R^2=0.98, respectively. At S/N = 3, the LOD was 2.0 pM DUL.

However, for nanoNIP-SWCNT@polytyramine, the R_{ct} did not change much with the logarithmic of the DUL concentration change (Figure S3c, Supplementary Information). The linear regression equation was $(R_{ct,0} - R_{ct,s})/R_{ct,0}$=0.01 (±0.004)/log [nM] × log c_{DUL} [nM] + 0.02 (±0.009). The sensitivity was 0.01 (±0.004)/log [nM] at S/N=3. The apparent imprinting factor, calculated as the ratio of slopes of the EIS calibration plots for nanoMIPs and nanoNIPs, was considerable, equaling IF = 7.5.

Moreover, the selectivity coefficients (α) for glucose and creatinine were 15 and 1.4, respectively. Advantageously, the EIS chemosensor was irresponsive to cholesterol in the same concentration range.

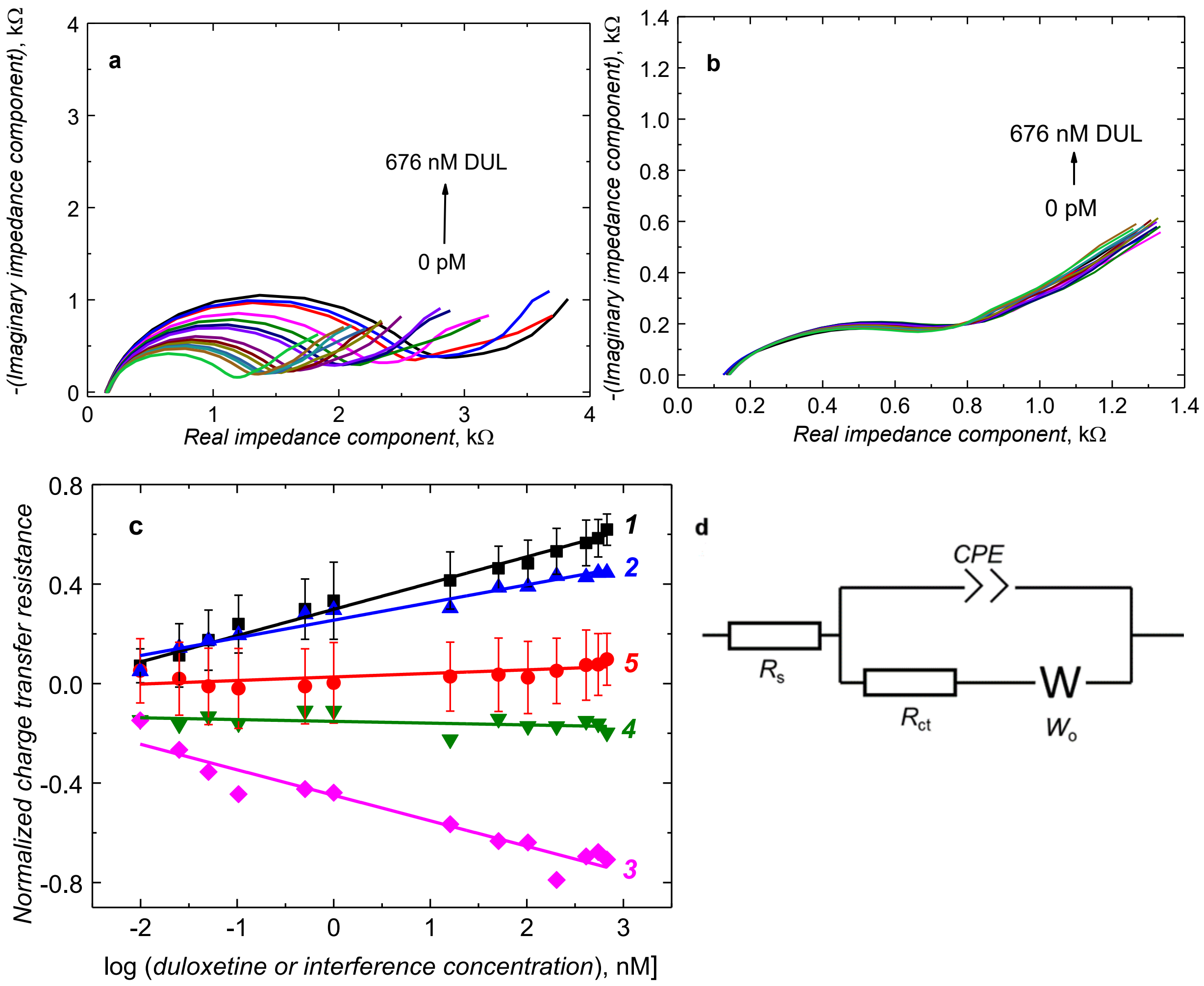

Figure S3. Nyquist plots for 2-mm diameter Au-disk electrodes coated with the SWCNTs@polytyramine films containing (a) nanoMIPs and (b) nanoNIPs after addition of DUL of different concentrations indicated at curves in 10 mM $K_3[Fe(CN)_6]$ and 10 mM $K_4[Fe(CN)_6]$, in 0.1 M PBS (pH=7.2) vs. Ag quasi-reference electrode. (c) The semilogarithmic calibration plot of the normalized charge transfer resistance against the DUL or interferences' concentration constructed using electrodes coated with the SWCNTs@polytyramine film containing (curves *1* - *4*) nanoMIPs and (curve 5) nanoNIPs. Plots 1, 2, 3, and 4 are respective calibration plots for DUL, creatinine, cholesterol, and glucose. (d) A modified Randles-Ershler equivalent circuit used to fit the EIS spectra.

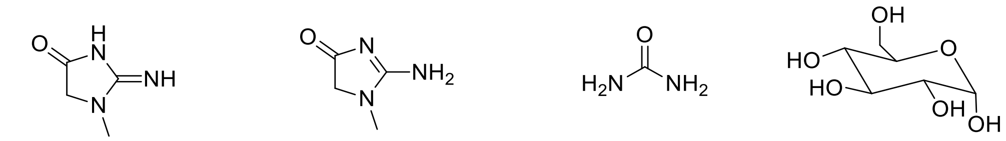

Imino tautomer of creatinine **Amino tautomer of creatinine** **Urea** **Glucose**

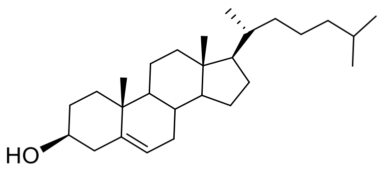

Cholesterol

Figure S4. Structural formulas of the creatinine tautomers, urea, glucose, and cholesterol interferences.

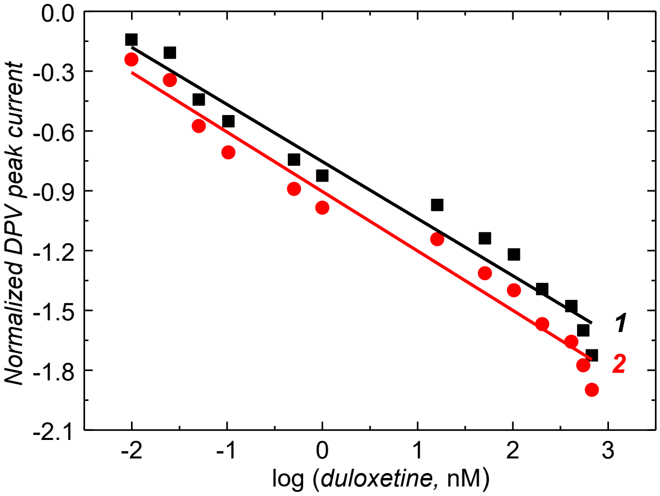

Figure S5. Calibration plots for DUL constructed using DPV normalized peak currents at electrodes coated with the SWCNT-polytyramine film containing nanoMIPs in 10 mM $K_3[Fe(CN)_6]$ and 10 mM $K_4[Fe(CN)_6]$ in 0.1 PBS (pH=7.2) vs. Ag quasi-reference electrode. Curves *1* and *2* represent calibration plots for DUL constructed with the electrode re-used after two months and initially used, respectively.

Table S3. Calculated changes of the Gibbs free energy (ΔG_{bind}) due to nanoMIP binding of the DUL analyte and selected interferences, experimental values of the imprinting factor (*IF*), and the selectivity coefficient (α) obtained from DPV experiments.

Compound	Experimental selectivity coefficient α	Calculated ΔG_{bind} (kJ/mol)
Duloxetine (DUL)	-	-201.96
Creatinine/tautomer of Creatinine	5.5	-55.54/-100.75
Cholesterol	-	-24.45
Urea	7.8	-68.20
Glucose	13.0	-36.30

S6. Modeling nanoMIPs cavity interactions with molecules of the DUL analyte and interferences

The analysis of the nanoMIPs intermolecular interactions with DUL revealed that oxygen atoms of the carbonyl of two molecules of MAA and one molecule of EGDMA formed strong hydrogen bonds with the nitrogen atom of the DUL amino group (length 1.87 to 3.02 Å). Moreover, the thiophene sulfur atom of DUL and the MAA hydroxyl formed a strong hydrogen bond (length of 2.18 Å) and electrostatic interaction (length of 2.74 Å). These interactions are responsible for the nanoMIP recognition of DUL. The medium-strength hydrogen bonds between the CH_3-NH_2^+ group of DUL and oxygen atoms of the carbonyls of other EGDMA and MAA molecules (lengths 2.54 to 2.82 Å) additionally strengthen the DUL sorption.

As a result, the 2-amino-1-methyl-2-imidazoline-4-one tautomer creates hydrogen bonds of NH_2 and CH_3 groups with MAA and EGDMA in the polymer matrix (length 1.84 to 2.96 Å) stronger than those formed by the 2-imino-1-methyl-2-imidazolidine-4-one tautomer. The urea molecule is located deep inside the cavity in its wall proximity, revealing the hydrogen bonds, as does the amino group of DUL. That results in forming hydrogen bonds of the urea molecule with one molecule of MAA and two molecules of EGDMA (lengths of 1.92 to 2.79 Å), but not as many bonds as DUL does. The glucose molecule is located deep inside the DUL-nanoMIP

cavity. Its hydroxyls form medium-strength hydrogen bonds with oxygen atoms of the MAA or EGDMA moieties (length 2.48 to 2.84 Å), responsible for the sorption. Moreover, water-supported four hydrogen bonds (length 2.24 to 3.19 Å) bind the glucose molecule inside the cavity. Two locations of a voluminous cholesterol molecule were analyzed, one with the OH group directed outside the cavity and the other with the OH group pointing towards the inside of the cavity. In both cases, the cholesterol molecule is positioned mainly outside the cavity. In one configuration, its OH group interactions with water help keep it inside the cavity. In the other configuration, forming the medium strength hydrogen bond of the MAA hydroxyl with the cholesterol molecule (length of 2.52 Å) can be responsible for the sorption. Both cases' analyses confirmed the above inference that cholesterol sorption is unlikely.

a

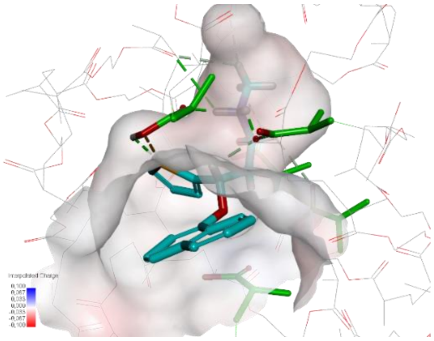

b

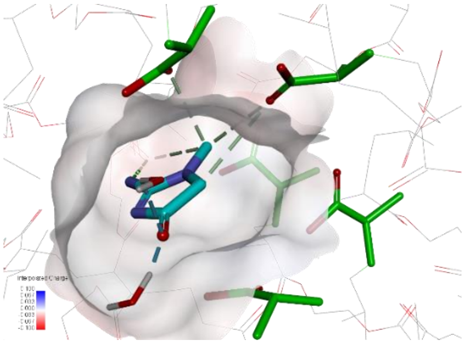

c

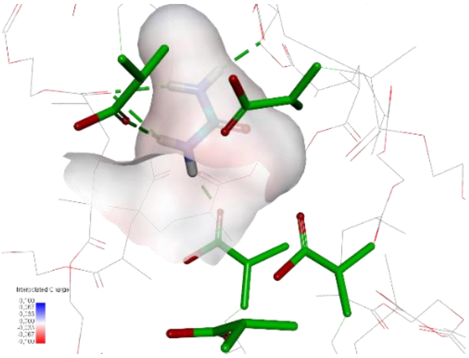

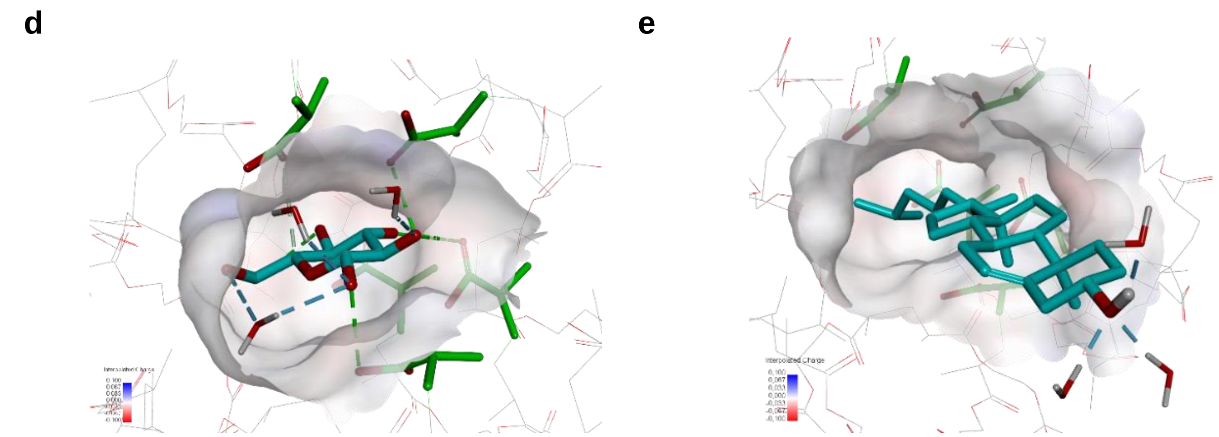

Figure S6. A simulated visualization of (a) the DUL analyte as well as (b) the creatinine 2-amino-1-methyl-2-imidazoline-4-one tautomer, (c) urea, (d) glucose, and (e) cholesterol interference molecule in the theoretically generated DUL-nanoMIP cavity.

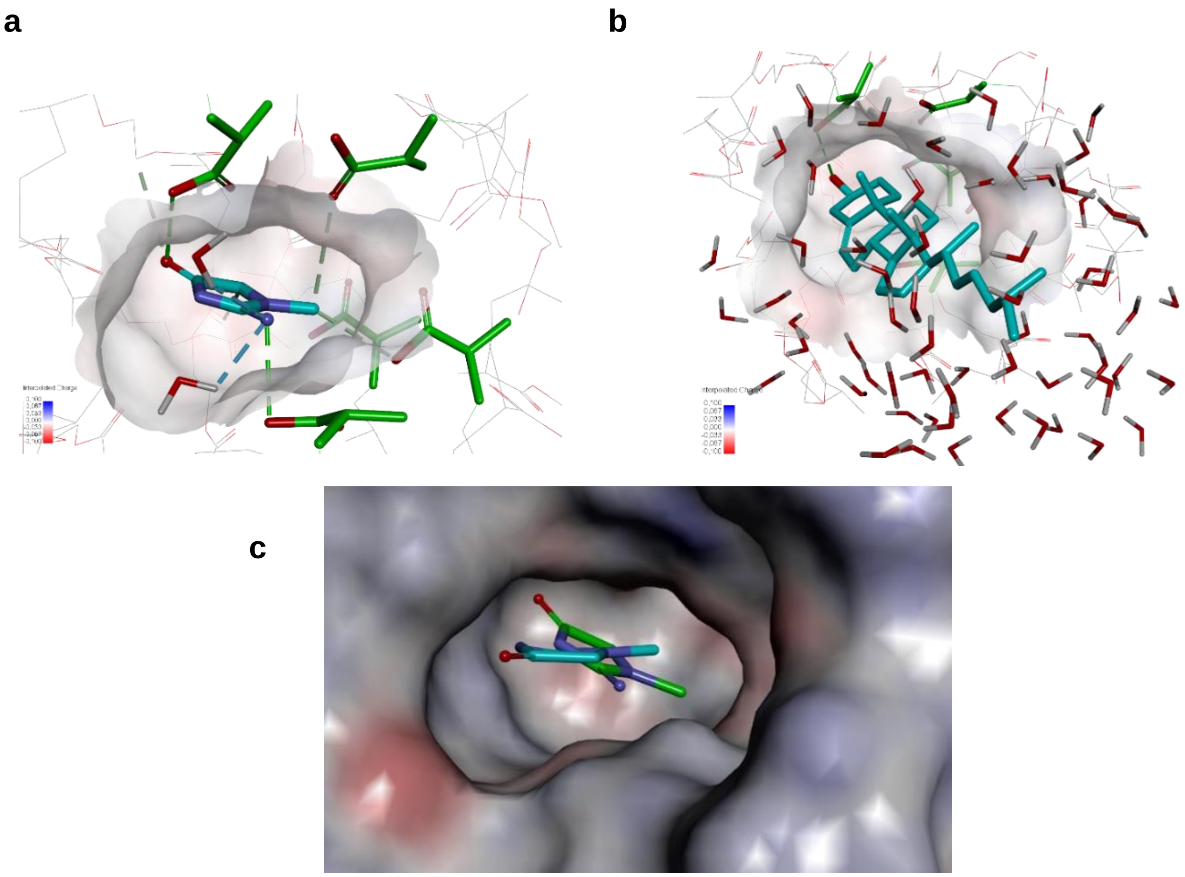

Figure S7. Computationally simulated visualization of (a) creatinine and (b) cholesterol interferences in the theoretically generated nanoMIP cavity. (c) Location of the 2-imino-1-methyl-2-imidazolidine-4-one (green) and 2-amino-1-methyl-2-imidazoline-4-one (turquoise) molecule in the cavity.

S7. DUL-nanoMIP selectivity to DUL metabolites

Different forms of cytochrome P450 can metabolize DUL, and structurally similar DUL derivatives can remain in equilibrium in biological samples. The devised and fabricated nanoMIPs may reveal affinities to all these derivatives. The simulation of sorption of DUL primary metabolites enables analyzing this issue. DUL is extensively excreted into the urine in the conjugated form. Its biotransformation is connected with hydroxylation of the naphthyl ring at either the 4-, 5- or 6- positions, followed by demethylation and (or) conjugation.[13] These data were complemented using the independent mathematical models employed in the ADMET program (ADMET Predictor version 10.0 software from Simulations Plus Inc.) The most frequently defined active metabolites, vis., DUL-1, DUL-1a, and DUL-1b, are chosen for the analysis (Figure S8, Supplementary Information).

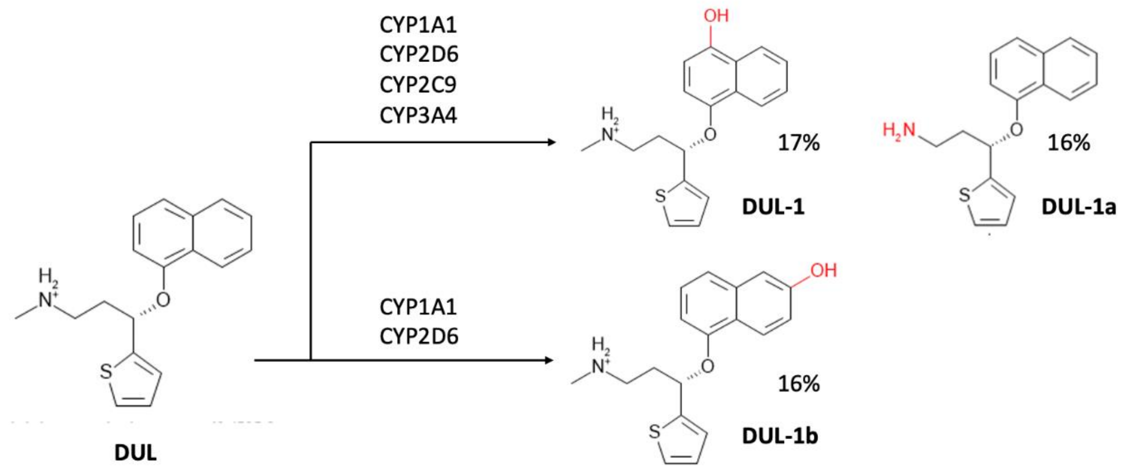

Figure S8. The phase I DUL metabolites chosen for the computational modeling.

The metabolites sorption was simulated according to the given procedure, and the final locations of the metabolites in the cavity are presented in Figure S9 in Supplementary Information. The metabolites are located in a similar place in the cavity as DUL, but the naphthyl ring of DUL-1b is turned, disturbing the interactions with the cavity.

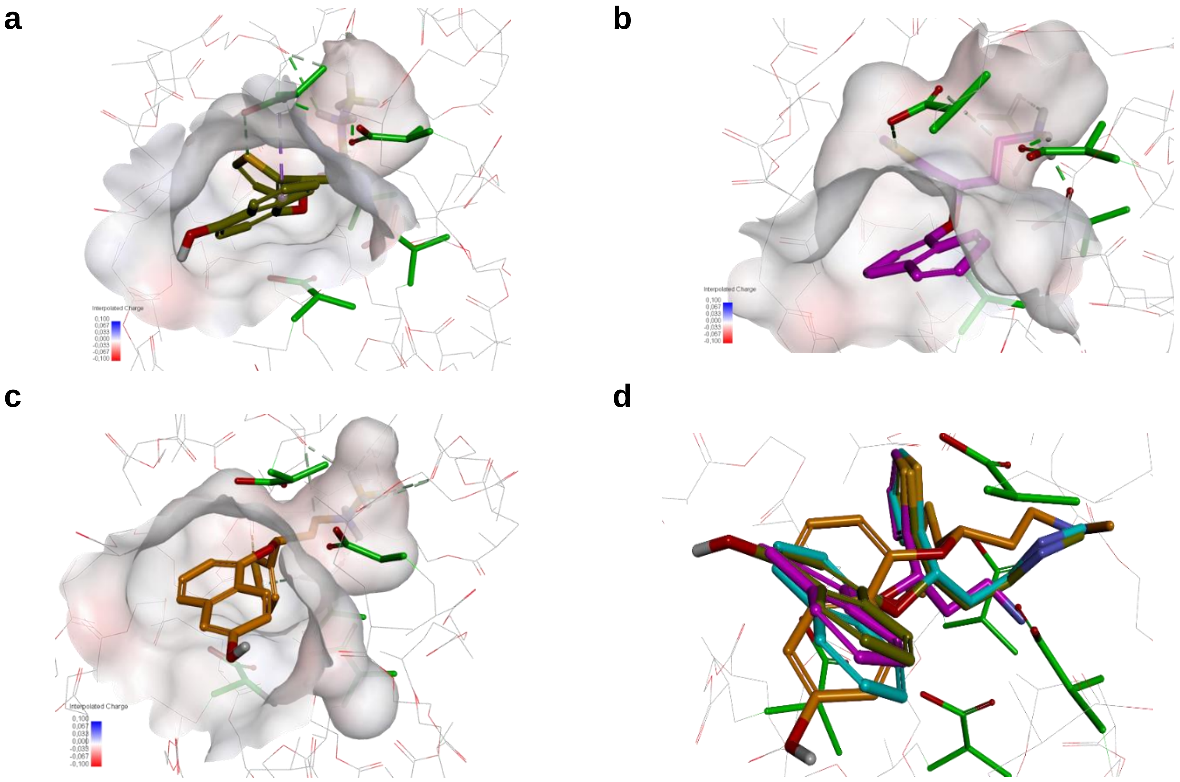

Figure S9. Simulated visualization of DUL metabolites in the DUL-nanoMIPs cavity. (a) DUL-1, (b) DUL-1a, (c) DUL-1b, and (d) superposition of DUL (turquoise) and its metabolites, vis. DUL-1 (dark green), DUL-1a (fuchsia), and DUL-1b (orange).

The DUL-1 metabolite is engaged in many hydrogen bonds with the MAA moiety's carboxyl or the polymer's EGDMA moiety's ester group. Oxygen atoms of the carbonyls of two MAA molecules and one oxygen atom of the EGDMA molecule form strong hydrogen bonds with nitrogen atoms of the amino group of DUL-1 (length 1.83 to 3.02 Å). Moreover, such bonds are formed between the thiophene sulfur atom of DUL-1 and the hydroxyl of the MAA moiety (length of 2.42 Å), but they are weaker than those for DUL. Furthermore, similar to DUL, there are weak hydrogen bonds between the methyl substituent of DUL-1 and the carbonyl's oxygen atoms of the EGDMA moiety (length 2.63 to 2.89 Å). Minor differences found in DUL-1 naphthyl ring orientations, compared to those in DUL, result in creating an additional π-sigma type interaction with the -CH_2- group of the MAA moiety (length of 3.99 Å). The respective calculated values of the Gibbs binding free energy change (ΔG_{bind}) of DUL metabolites, vis., DUL-1, DUL-1a, and DUL-1b, are -147.61, -11.05, and 34.89 kJ/mol,

respectively. Apparently, only DUL-1 reveals some affinity to nanoMIP, but this affinity is much lower than that of DUL. To sum up, the devised chemosensor can be used for DUL determination in the presence of its metabolites because these should not interfere with this determination.

References

(1) Al-Qahtani, S. D. Potentiometric Determination of Duloxetine Hydrochloride Using Coated Wire Electrode in Pure and Pharmaceutical Preparations. *Asian J. Pharm.* **2017**, *11*, S854–S857.

(2) Ammar, R. A.; Otaif, H.; Al-Warthan, A. Quantitative Determination of Duloxetine Hydrochloride in Pharmaceuticals and Urine Using Prepared Ion-Selective Membrane Electrode. *Int. J. Electrochem. Sci.* **2012**, *7*, 4369–4380.

(3) Hassanein, A. M.; Moharram, Y. I.; Oraiby, N. F.; Ebied, S. E. Trace Determination of Duloxetine HCl in Formulation and Spiked Human Serum at a Carbon Paste Electrode. *Am. J. Anal. Chem.* **2017**, *08*, 708–725.

(4) Attia, A. K.; Rashed, N. S.; Mohamed, O. A.; Kamal Attia, A. Voltammetric Assay of Duloxetine Hydrochloride at Carbon-Based Electrode Modified by Titanium Dioxide Nanoparticles Enriched with Multi-Walled Carbon Nanotubes. *TrAC - Trends Anal. Res.* **2018**, *1*, 1–6.

(5) Alarfaj, N. A.; Ammar, R. A.; El-tohamy, M. F. Disposable Screen-Printed Sensors for Determination of Duloxetine Hydrochloride. *Chem. Cent. J.* **2012**, *6*, 1–13.

(6) Prabu, S. L.; Shahnawaz, S.; Dinesh Kumar, C.; Shirwaikar, A. Spectrofluorimetric Method for Determination of Duloxetine Hydrochloride in Bulk and Pharmaceutical Dosage Forms. *Indian J. Pharm. Sci.* **2008**, *70*, 502–503.

(7) *BIOVIA, Dassault Systèmes Discovery Studio Modeling Environment., Release 2017.*; San Diego: Dassault Systèmes, 2016.

(8) Frisch, M. J.; Trucks, G. W.; Schlegel, H. B.; Scuseria, G. E.; Robb, M. A.; Cheeseman, J. R.; Scalmani, G.; Barone, V.; Petersson, G. a.; Nakatsuji, H.; Li, X.; Caricato, M.; Marenich, A. V.; Bloino, J.; Janesko, B. G.; Gomperts, R.; Mennucci, B.; Hratchian, H. P.; Ortiz, J. V.; Izmaylov, A. F.; Sonnenberg, J. L.; Williams; Ding, F.; Lipparini, F.; Egidi, F.; Goings, J.; Peng, B.; Petrone, A.; Henderson, T.; Ranasinghe, D.; Zakrzewski, V. G.; Gao, J.; Rega, N.; Zheng, G.; Liang, W.; Hada, M.; Ehara, M.; Toyota, K.; Fukuda, R.; Hasegawa, J.; Ishida, M.; Nakajima, T.; Honda, Y.; Kitao, O.; Nakai, H.; Vreven, T.; Throssell, K.; Montgomery Jr., J. a.; Peralta, J. E.; Ogliaro, F.; Bearpark, M. J.; Heyd, J. J.; Brothers, E. N.; Kudin, K. N.; Staroverov, V. N.; Keith, T. a.; Kobayashi, R.; Normand, J.; Raghavachari, K.; Rendell, a. P.; Burant, J. C.; Iyengar, S. S.; Tomasi, J.; Cossi, M.; Millam, J. M.; Klene, M.; Adamo, C.; Cammi, R.; Ochterski, J. W.; Martin, R. L.; Morokuma, K.; Farkas, O.; Foresman, J. B.; Fox, D. J. *Gaussian 16, Inc.*; Wallingford CT, USA, 2016.

(9) Breneman, C.; Wiberg, K. Determining Atom-Centered Monopoles from Molecular

Electrostatic Potentials. The Need for High Sampling Density in Formamide Conformational Analysis. *J. Comput. Chem.* **1990**, *11*, 361–373.

(10) Ryckaert, J.-P.; Ciccotti, G.; Berendsen, H. J. C. Numerical Integration of the Cartesian Equations of Motion of a System with Constraints: Molecular Dynamics of n-Alkanes. *J. Comput. Phys.* **1977**, *23*, 327–341.

(11) Martinez, L.; Andrade, R.; Birgin, E. G.; Martinez, J. M. Packmol: A Package for Building Initial Configurations for Molecular Dynamics Simulations. *J. Comput. Chem.* **2009**, *30*, 2157–2164.

(12) Jorgensen, W. L.; Chandrasekhar, J.; Madura, J. D.; Impey, R. W.; Klein, M. L. Comparison of Simple Potential Functions for Simulating Liquid Water. *J. Chem. Phys.* **1983**, *79*, 926–935.

(13) Lantz, R. J.; Gillespie, T. A.; Rash, T. J.; Kuo, F.; Skinner, M.; Kuan, H.-Y.; Knadler, M. P. Metabolism, Excretion, and Pharmacokinetics of Duloxetine in Healthy Human Subjects. *Drug Metab. Dispos.* **2003**, *31*, 1142–1150.
